# Prevalence of Alcohol Use Disorders and Their Association with Sociodemographic Determinants and Depression/Anxiety Disorders in a Representative Sample of the Greek General Population

**DOI:** 10.1155/2020/4841050

**Published:** 2020-02-10

**Authors:** Stefanos Bellos, Petros Petrikis, Meni Malliori, Venetsanos Mavreas, Petros Skapinakis

**Affiliations:** ^1^Department of Psychiatry, Faculty of Medicine, School of Health Sciences, University of Ioannina, Ioannina, Greece; ^2^National and Kapodistrian University of Athens, Eginition Hospital, Athens, Greece

## Abstract

**Background:**

Country-level epidemiological data about alcohol-related problems is useful for planning prevention and treatment services. Heavy Alcohol Consumption (HAC) and Alcohol Use Disorder (AUD) are two syndromes of alcohol-related problems that have been recognized worldwide. Study of the epidemiological determinants of HAC and AUD in different sociocultural contexts could inform hypotheses about the etiology or the consequences of alcohol-related problems.

**Objectives:**

We assessed the prevalence and associations of HAC and AUD with sociodemographic variables adjusting for common mental disorders in a representative sample of the general population of Greece (*N* = 4894 participants). The period of data collection just preceded the emergence of the financial crisis in Greece.

**Results:**

The majority of the population did not report HAC, AUD or abstinence from alcohol. HAC was reported by 12.7% (95% CI: 11.8–13.6) of the population while 3.1% (95% CI: 2.7–3.6) met criteria for AUD. Younger age, divorce, lower educational level, living in an urban area, physical health problems, and smoking were associated with a higher prevalence of both conditions. Presence of severe financial difficulties and never married family status were associated with a higher prevalence of HAC but not AUD. HAC was associated with nonspecific psychiatric morbidity while AUD was associated with more specific psychiatric disorders. *Conclusion/Importance*. Both alcohol-related problems are frequent in the general population and have common and distinct determinants. The comparison between the findings of our study and those of similar studies during or after the period of financial austerity in Greece, would offer the opportunity to assess the possible effects of changes in the economical context in the determinants of alcohol-related problems.

## 1. Introduction

Until recently, two main types of Alcohol-related problems, namely alcohol “abuse” and “dependence,” had been recognized by the ICD-10 and DSM-IV. As there has been evidence that those two types of alcohol-related problems lie on the same continuum [1], in the recent revision of DSM-5 abuse and dependence have been merged and replaced by the common diagnosis of “Alcohol Use Disorder” (AUD). Nevertheless, there is some evidence that alcohol abuse may present different associations with sociodemographic and mental health determinants compared to alcohol dependence or AUD [2–8].

Most of the national community studies that have examined those two types of Alcohol-related problems [1, 9–11] limit their results to patients endorsing diagnostic criteria for alcohol “dependence” or “abuse.” However, diagnostic criteria may be subjected to change over time [12] and may have restricted validity in a cross-cultural context [13, 14]. Another option for defining different types of alcohol-related problems could by the application of self-assessment screening instruments, which would be more reasonable from a public health perspective. AUDIT (Alcohol Use Disorders Identification Test) is the most trans-culturally validated instrument for measuring alcohol-related problems in the general population and can reliably discriminate between Heavy Alcohol Consumption (HAC) (3 first AUDIT-items) and Alcohol Use Disorder (AUD) (all AUDIT-items) [15–17].

HAC constitutes an equivalent but broader definition of alcohol “abuse” which presents less cross-cultural variability as it is describes only the total amount of alcohol consumed and does not take into account its effects in behavior, which may be more context-dependant. Indeed, in studies originating from countries that have a more permissive culture over alcohol use, the assessment of alcohol consumption may be more valuable from a preventive perspective since many heavy alcohol consumers, may not fulfill criteria for dependence due to the social acceptance of some alcohol.

To our knowledge, no other epidemiologic survey in Greece has recruited a nationally representative sample to assess HAC and AUD. Previous Greek studies cannot reach valid conclusions on the prevalence of HAC and AUD, as they recruited convenient rather than representative samples [18], and the studies which attempted to recruit general populations samples have been outdated [19, 20]. Our findings acquire an additional interest as they have been collected just before the initiation of the financial crisis in Greece and could be used as a valid pre-crisis comparator for current or future studies originating from Greece that would attempt to assess the possible effect of the financial crisis on alcohol-related problems and their determinants.

The aims of the current study were:

1. To estimate the prevalence of alcohol abstinence, moderate consumption, HAC, and AUD,
2. To assess the associations of HAC and AUD with socioeconomic determinants and common mental disorders as well the differences in those associations that the two types of alcohol misuse present.

## 2. Methods

### 2.1. General Description of the Dataset

Our dataset is a part of the Psychiatric Morbidity Survey in Greece, a cross-sectional study carried out using a nationally representative sample of the adult population (18–70 years). Data collection was conducted between September 2009 and February 2010. Eligible for participation were all adults living in households in all areas of Greece. We covered both mainland and insular regions with the exception of Crete (due to budget limitations).

### 2.2. Sampling Procedure—Data Collection

Our sample was collected using a three-stage sampling design. At the first stage unified city blocks based on the 2001 census survey of Greece were selected. Households within the selected areas at the second stage and individuals within the households at the third stage were selected, following a systematic random sampling procedure (e.g., last birthday method). Finally, 4894 adults accepted to participate in our study from an initial sample of 9800 individuals aged 18−70 years (response rate = 54%). Differences between the sample and the 2001 census population data were small, confirming that way the representativeness of our sample. Data was collected by trained lay-interviewers using measurement instruments in a computerized form. A full detail of the study design, sampling procedures, sampling distribution, and data collection are available in our previous publications [23].  Table 1 presents the basic description of the sample.

### 2.3. Measurement of Psychiatric Morbidity

Psychiatric morbidity was assessed with a fully structured psychiatric interview, the “Revised Clinical Interview Schedule” (CIS-R) [24, 25]. The interview was administered by trained lay interviewers using a computerized version that has been found to be comparable with the face-to-face interview [26].

The CIS-R assesses the frequency, duration, and severity of 14 common psychiatric symptoms. Each symptom is scored from 0 to 4, allowing for a dimensional measure of psychiatric morbidity by summing-up all 14 symptoms. A total CIS-R score ≥ 12 usually represents “clinically significant” psychiatric morbidity and a score of 18 or more corresponds to more “severe case” [24]. Additional questions, including the assessment of functioning impairment, enable the diagnosis of five common mental disorders (depressive episode, generalized anxiety disorder, all specific phobias combined, panic disorder, and obsessive-compulsive disorder) according to the ICD-10 research diagnostic criteria (categorical measure). The diagnosis of “mixed anxiety and depression disorder” was assigned to participants with CIS-R score ≥  12 who did not meet ICD-10 diagnostic criteria for any other specific psychiatric disorder. The Greek version of the CIS-R has been validated and its psychometric properties have been reported [27].

### 2.4. Assessment of Alcohol-Related Problems

Alcohol-related problems were assessed with AUDIT [15] which has been previously validated in the Greek population [18]. The AUDIT questions are referred to the period of the last year, so the presented prevalence estimates could be considered as “last-year prevalence”. In the present study we measured two different dimensions or alcohol-related problems:

1. Heavy alcohol Consumption (HAC), by summing up the first three questions of the AUDIT (average alcohol consumption per session, frequency, binge drinking) to calculate the AUDIT-C subscale with a range of scores from 0 to 12 [2]. To define HAC we used the cut points suggested by Aalto et al. [28] (6 or more for men and 4 or more for women).
2. Alcohol Use Disorder (AUD), by using the total AUDIT score ranging from 0 to 40. Generally, the researchers who initially developed the instrument suggest a cut-off of 15 in total AUDIT-score as indicative for the presence of AUD [17], but there is some evidence that the cut-offs are lower in women [29]. Therefore we used cut-offs of 15 for men and 11 for women to define AUD.

### 2.5. Assessment of Socio-Demographic and Health-Related Variables

Information about sex, age, marital status, employment status, self-reported financial difficulties, and educational qualifications were obtained from the participants. Regarding employment status, we distinguished between unemployment (i.e., the participant did not do any paid work but looked for work in the past 4 weeks) and 3 economic inactivity groups: (a) looking after the house, (b) retired, and (c) a residual category of “other economically inactive” (including students, those unable to work, etc.). Participants were also asked if they suffer from any chronic and severe medical conditions (e.g., coronary heart disease, stroke, chronic lung diseases, diabetes, malignancy, etc.) using a predetermined list of these conditions. Regarding smoking, participants were defined as “smokers” if they were reported smoking everyday at least 2 or more cigarettes on average during the past month.

### 2.6. Statistical Analysis

Data were weighted to account for the complex sampling design and non-response. We used the survey commands in Stata version 12.0 to calculate prevalence estimates and 95% Confidence Intervals (CI). These commands take into account the complex sampling design and compute robust standard errors. Associations between HAC or AUD with common mental disorders and sociodemographic variables were examined using odds ratios and their 95% CI, which were calculated with a series of adjusted logistic regression models using the survey commands in Stata 12.0. All evaluations of statistical significance are based on two-sided tests using the 5% level of significance.

## 3. Results

### 3.1. Prevalence of Heavy Alcohol Consumption (HAC) and Alcohol Use Disorder (AUD)

The majority of the population did not report HAC, AUD or abstinence from alcohol. The last year prevalence of HAC in our sample was estimated at 12.7% (95% CI: 11.8–13.6) and of AUD at 3.1% (95% CI: 2.7–3.6). Both types of alcohol-related problems were much more common in men compared to women (*p* < 0.05 ) (Table 2). The total distribution of AUDIT and AUDIT-C scores in our sample is presented in Table 3.

### 3.2. Association of HAC and AUD with Sociodemographic Factors

HAC was more prevalent in never-married and divorced individuals compared to married, in residents from rural and semi-urban places compared to those of urban origin, in individuals reporting more financial difficulties, in lower educated participants compared to higher, and in smokers. Unemployment was weakly associated with higher prevalence of HAC and economic inactivity (retired, looking after home) with lower prevalence of HAC, comparing to workers after controlling for other sociodemographic variables (Table 4).

Regarding AUD, higher prevalence was observed in individuals with lower educational level, smokers, being divorced, and living in a rural region, similar to the associations observed with HAC. Contrary to HAC, AUD was not statistically associated with being never-married, the presence of financial difficulties and semi-urban residency but there was a strong association with the presence of physical illness. Similarly to the case of HAC, there were no significant associations of AUD with unemployment and economic inactivity status (Table 5).

### 3.3. Association of HAC and AUD with Common Mental Disorders

AUD was more strongly associated with psychiatric morbidity compared to HAC. HAC was significantly associated only with mixed depressive-anxiety disorders, which resembles an undifferentiated form of clinical significant symptomatology, not assigned to any specific psychiatric disorder (Table 6). AUD was associated with Anxiety disorders [Panic Disorder (age- and sex-adjusted (OR = 4.35, 95% CI: 2.02–9.38), Phobic Disorder (OR = 3.34, 95% CI: 1.6–6.8), GAD (OR = 2.96, 95% CI: 1.59–5.54)], and Depression (OR = 2.24, 95% CI: 1.01–4.95). Interestingly, the association of AUD with Depression was not statistically significant after taking into consideration other sociodemographic and health-related factors [OR = 1.54, 95% CI: 0.67–3.54] (Table 7).

## 4. Discussion

### 4.1. Main Findings

Moderate, nonproblematic, drinking is the most common alcohol-related behavior in the Greek general population as the vast majority of the Greek alcohol users did not face AUD. More that 10% of the general population uses alcohol heavily and about 3% face AUD. Both types of alcohol-related problems are more common in males and in younger individuals. However the majority of the population uses alcohol in a nonproblematic way. Divorce, lower educational level, living in an urban area, and smoking were associated with a higher prevalence of both conditions independent of other covariates. Presence of severe financial difficulties and never-married family status were associated with a higher prevalence of HAC but not AUD. Physical health problems were associated with AUD but not HAC. Generally, both conditions have similar associations with socioeconomic and health-related variables but the observed associations were stronger for AUD. HAC was associated with nonspecific psychiatric morbidity while AUD was associated with more specific psychiatric disorders.

### 4.2. Interpretation of the Results

The different associations with mental health problems between HAC and AUD highlight an idiosyncrasy of Greek culture over alcohol, which may be used in larger quantities mainly as a nutritional element rather than as an intoxicant or for self-medicating mental health conditions [30]. Τhe permissive culture that the Mediterranean population exhibits on alcohol consumption is not associated with higher prevalence of alcohol use disorders comparing to societies with relatively lower permissive levels. This fact indicate that the level of acceptance of a substance is associated with higher prevalence of heavy use but not necessarily with higher prevalence of substance related problematic behaviors.This approach is also in line with studies supporting that HAC may not necessarily lead to the development of mental health problems, unless other alcohol-related problematic behaviour occurs [4, 6, 7], implying that heavy alcohol consumption is a necessary but not sufficient prerequisite condition for the development of AUD.

### 4.3. Comparison with Other Studies

In most large population studies on alcohol related behavior, AUD is measured using the diagnostic criteria for “abuse” and “dependence,” while in our study we defined HAC and AUD using a cross-cultural validated screening instrument, for reasons presented in the introduction. Thus, only indirect comparisons on prevalence of AUD can be made, assuming that in our study AUD resemble the combination of the formerly considered diagnoses of “abuse” and “dependence.” Taken into account this assumption, the estimated prevalence of AUD in the Greek population is slightly lower compared with that measured from US studies [1, 11] and similar to the estimations from Australian [9, 10] and European studies [31]. In general, there is a significant variation on the estimated prevalence of AUD [32, 33] that can be explained as a result of true variation due to the different culture over alcohol consumption across countries or of different methodologies which are used to define AUD across studies.

AUD cooccur with mental health problems at much higher levels than chance, in concordance with other studies [34, 35]. The observed associations are stronger for Anxiety disorders comparing to Depression, similar to other community studies originated from populations that alcohol abstention is infrequent [9, 36]. Moreover, the association between AUD and Anxiety disorders is less influenced by the presence of sociodemographic determinants comparing to the association with Depression, implying a more distinct association of AUD with Anxiety disorders in concordance with large epidemiological studies [34, 37, 38].

Regarding the associations with sociodemographic variables, the following factors are undoubtedly associated with Alcohol-related problems, in concordance to the findings of our study: (1) male sex, (2) younger age, (3) smoking, with a dose-related reciprocal association [39–42], and (4) divorce and never-married condition [9, 43, 44]. However, widowhood is inversely associated with HAC but positively associated with AUD, in our study. Taking into account the discrepancy in studies about the association between widowing and HAC [45, 46] it is very likely that this association is mediated by other vulnerability factors like copying strategies/styles [47] and factors that precipitate the marriage life [46].

Regarding the association between alcohol-related problems and the variables of financial difficulties and unemployment our findings conclude appreciable positive associations which are significantly modified by the presence of other covariates. This finding is in concordance with findings from other studies which support that although unemployment is a cause of increased alcohol consumption [48–50] the interaction caused by other determinants, like the duration of unemployment [51, 52], the economic environment in which unemployment is taking place [53], the educational level, socioeconomic status, and previous drinking history of the individual [54, 55], can significantly modify the association between unemployment and alcohol-related problems. Similarly, although income is linearly associated with alcohol consumption [41], economic hardship has been associated with increased odds for abstinence as well as for heavy drinking [56], probably due to the self-medicating properties of alcohol [57, 58].

Moreover, higher educational level is strongly associated with decreased probability for HAC and AUD in our sample, in partial agreement with most of the studies concluding that lower educational level is associated with more alcohol-related problems [59–61] but not with HAC [62, 41] and in dissonance with studies which concluded no association between alcohol-related problems and educational level [9]. It is more likely that the association between educational level and alcohol-related behaviour is not universal and depends on the characteristics of the country or the culture over alcohol consumption [63], as educational level may play a moderating role between psychological and environmental factors predisposing to alcohol problems [64, 65].

Regarding place of residency, in our study, rural residency is associated with higher prevalence of AUD and HAC in concordance with studies supporting that although rural residence is protective against any alcohol consumption, is a risk factor for the development of AUD among those who make the decision to drink [66, 54]. It is possible that the heterogeneity that rural areas present in social, cultural, and policy-level factors effect drinking patterns [66].

### 4.4. Limitations

Our study is cross-sectional and we cannot make conclusions about the temporal association between Alcohol-related problems and the studied variables. Therefore we cannot resolve questions regarding the underlying causal mechanisms, as inverse causality (e.g., AUD to be the prerequisite factor and not the result) may be true.

Moreover, despite the refined methodology that we followed for the sample selection, selection biases cannot be excluded, as the response rate was relatively low. Such biases may have lead to underestimation of AUD in our study, as it may be more possible for sufferers from AUD and participants with lower socioeconomic status to refuse to participate in community studies.

Another limitation, common in studies of the association between mental health and Substance Use Disorders, is that the diagnosis of current mood or anxiety disorders among active substance abusers may be complicated by the fact that intoxication or withdrawal symptoms may resemble the symptoms of mood and anxiety disorders.

Finally, AUDIT is a screening tool and not a diagnostic instrument and the cut-offs used for assessing alcohol abuse and dependence in different studies are heterogeneous. This might have influenced the comparability of the estimated prevalence in our study with other epidemiologic studies in which different cut-off value of AUDIT or diagnostic schedules were used to assess alcohol use problems. But, the comparability of the observed associations of alcohol use problems with sociodemographic variables and mental health status is expected to slightly be influenced by the different ways that alcohol use problems are defined.

## 5. Conclusion

Evidence from psychiatric epidemiology studies suggests that although certain risk factors (e.g., younger age, male sex, divorce/never-married, smoking) are consistently associated with alcohol-related problems, the pattern of association between AUD and depression/anxiety disorders and other socioeconomic determinants as well as the underlying mechanisms, may significantly vary across countries [67, 68]. This variation is probably the result of the effect of different cultural and policy factors on alcohol-related behavior and restricts the cross-cultural generalisability of the large US and Australian national studies [1, 9–11]. Therefore, there is a need to acquire culture-specific data in order to better comprehend the cross-national variations in the determinants of Alcohol-related problems contributing to the formulation of effective alcohol policies, taking into account the uniqueness of each culture in alcohol consumption.

Finally, the current survey was conducted in 2009 before the continuing last years' economic recession in Greece. There is some evidence that economic crisis has increased mental health disorders [69, 70] and is expected to further increase abstinence as well as heavy alcohol use [21, 22]. These changes are expected to diminish the possible positive outcomes of moderate alcohol use [71] and, thereof, cause modification in the associations of AUD with psychosocial determinants and mental health disorders. From that perspective, a pre and postrecession comparison of the prevalence of AUD and their determinants in Greece would enrich our knowledge about the effects of the economic downturns in alcohol-related problems.

## Figures and Tables

**Table 1:** Descriptive statistics of the sample (*N* = 4894).

	*N* (%)
*Sex*	
Male	2425 (49.6%)
Female	2469 (50.4%)

*Age group*	
18–29	1226 (25.1%)
30–39	1032 (21.1%)
40–49	934 (19.1%)
50–59	802 (16.4%)
60–70	900 (18.4%)

*Marital status*	
Married	2995 (61.2%)
Never-married	1446 (29.6%)
Divorced	240 (4.9%)
Widowed	213 (4.3%)

*Education*	
None/primary	926 (18.9%)
Lower secondary	797 (16.3%)
Upper secondary	2348 (48.0%)
Technical	439 (9%)
University	384 (7.8%)

*Employment status*	
Fully employed	2917 (59.6%)
Looks after home	691 (14.1%)
Unemployed	184 (3.8%)
Retired	577 (11.8%)
Other	525 (10.7%)

*Urbanicity*	
Urban	2682 (54.8%)
Semi-urban	607 (12.4%)
Rural	1605 (32.8%)

*Presence of financial difficulties*	
No	3283 (67.1%)
Yes	1611 (32.9%)

*Chronic diseases*	
No	4234 (86.5%)
Yes	659 (13.5%)

*Smoking*	
No	2955 (60.4%)
Yes	1937 (39.6%)

*Mental health disorders*	
Depression	142 (2.9%)
GAD	201 (4.1)
Panic disorder	92 (1.9%)
OCD	83 (1.7%)
Phobic disorders	137 (2.8%)
Mixed anxiety depressive disorder	131 (2.7%)

*CIS-R score*	
0–5	3484 (71.1%)
6–11	722 (14.7%)
12–17	332 (6.7%)
≥18	356 (7.2%)

**Table 2:** Heavy alcohol consumption and alcohol use disorder in a national representative sample of the Greek population (*N* = 4894).

Age group	18–34		35–44		55–70		Total	
	*N*	% (95% *CI*)	*N*	% (95% *CI*)	*N*	% (95% *CI*)	*N*	% (95% *CI*)
*Heavy alcohol consumption*								
Males (*AUDIT-C scores ≥6*)	149	16.7% (14.2–19.1)	185	20.1% (17.5–22.7)	77	12.6% (10.0–15.2)	411	16.9% 15.5–18.4)
Females (*AUDIT-C scores ≥4*)	103	11.9% (97–14)	80	8.5% (67–10.3)	27	4.1% (2.6–5.6)	210	8.5% (7.4–9.6)

Whole sample	252	14.3% (12.7–15.9)	265	14.3% (12.7–15.8)	104	8.2% (6.7–9.7)	621	12.7% (11.8–13.6)

*Alcohol use disorders*								
Males (*AUDIT score ≥15*)	45	5.0% (3.6–6.5)	57	6.2% (4.6–7.8)	22	3.6% (2.1–5.1)	124	5.1% (4.2–6.0)
Females (*AUDIT score ≥11*)	16	1.8% (0.9–2.7)	12	1.3% (0.6–2.0)	2	0.3% (0.1–0.7)	30	1.2% (0.8–1.6)

Whole sample	61	3.5% (2.6–4.3)	69	3.7% (2.9–4.6)	24	1.9% (1.1–2.6)	154	3.1% (2.7–3.6)

All comparisons are statistically significant (*p* < 0.05, Pearson chi-square) between “males” and “females” as well as younger (“18–34” & “35–54”) and older (“55–70”) participants. *N* = actual Number of observations % = weighted percentage

**Table 3:** AUDIT-C (alcohol consumption) and total AUDIT (alcohol use disorders) scores distribution in a national representative sample of 4894 Greek participants.

	Age group						Total	
	18–34		35–54		55–70			
	*N*	% (95% *CI*)	*N*	% (95% *CI*)	*N*	% (95% *CI*)	*N*	% (95% *CI*)
AUDIT-C scores								
*Males*								
0 (abstainers)	96	10.7% (*8.7*–*12.8*)	99	10.8% (*8.8*–*12.8*)	113	18.5% (*15.4*–*21.6*)	308	12.7% (*11.3*–*14*)
1–3 (moderate)	431	48.2% (44.9–51.4)	432	47% (43.8–50.2)	274	44.8% (40.9–48.8)	1137	46.9% 44.9–48.9)
4-5 (moderate to heavy)	219	24.5% (21.6–27.3)	203	22.1% (19.4–24.8)	147	24.1% (20.7–27.5)	569	23.5% (21.8–25.2)
6–12 (heavy alcohol consumption)	149	16.7% (14.2–19.1)	185	20.1% (17.5–22.7)	77	12.6% (10.0–15.2)	411	16.9% (15.5–18.4)

*Females*								
0 (abstainers)	214	24.6% (21.8–27.5)	306	32.6% (29.6–35.6)	393	59.5% (55.8–63.3)	913	37% (35.1–38.9)
1-2 (moderate)	450	51.8% (48.5–55.1)	478	50.9% (47.7–54.1)	210	31.8% (28.3–35.4)	1138	46.1% (44.1–48.1)
3 (moderate to heavy)	102	11.7% (9.6–13.9)	76	8.1% (6.3–9.8)	30	4.5% (3.0–6.1)	208	8.4% (7.3–9.5)
4–12 (heavy alcohol consumption)	103	11.9% (97–14)	80	8.5% (67–10.3)	27	4.1% (2.6–5.6)	210	8.5% (7.4–9.6)

*Whole sample*								
Abstainers	310	17.6 % (15.8–19.4)	405	21.8% (19.9–23.7)	506	39.8% (37.1–42.5)	1221	24.9% (23.7–26.2)
Moderate	881	49.9% (47.6–52.3)	910	49% (46.7–51.2)	484	38.1% (35.4–40.8)	2275	46.5% (45.1–47.9)
Moderate to heavy	321	18.2% (16.4–20)	279	15% (13.4–16.6)	177	13.9% (12–15.8)	777	15.9% (14.9–16.9)
Heavy alcohol consumption	252	14.3% (12.7–15.9)	265	14.3% (12.7 –15.8)	104	8.2% (6.7–9.7)	621	12.7% (11.8–13.6)

Total AUDIT score								

*Males*								
0–7 (no problems)	701	78.3% (75.6–81.0)	733	79.8% (77.2–82.4)	529	86.6% (83.9–89.3)	1963	80.9% (79.4–82.5)
8–14 (some problems)	149	16.6% (14.2–19.1)	129	14% (11.8–16.3)	60	9.8% (7.5–12.2)	338	13.9% (12.6–15.3)
15–40 (alcohol use disorders)	45	5.0% (3.6–6.5)	57	6.2% (4.6–7.8)	22	3.6% (2.1–5.1)	124	5.1% (4.2–6.0)

*Females*								
0–4 (no problems)	778	89.5% (87.5–91.6)	873	92.9% (91.2–94.5)	638	96.7% (95.3–98.0)	2289	92.7% (91.7–93.7)
5–10 (some problems)	75	8.6% (6.8–10.5)	55	5.9% (4.3–7.4)	20	3% (1.7–4.3)	150	6.1% (5.1–7.0)
11–27 (alcohol use disorders)	16	1.8% (0.9–2.7)	12	1.3% (0.6–2.0)	2	0.3% (0.1–0.7)	30	1.2% (0.8–1.6)

*Whole sample*								
No problems	1479	83.8% (82.1–85.6)	1606	86.4% (84.8–88)	1167	91.8% (90.3–93.3)	4252	86.9% (85.9–87.8)
Some problems	224	12.7% (11.1–14.3)	184	9.9% (8.5–8.8)	80	6.3% (5–7.6)	488	10% (9.1–10.8)
Alcohol use disorders	61	3.5% (2.6–4.3)	69	3.7% (2.9–4.6)	24	1.9% (1.1–2.6)	154	3.1% (2.7–3.6)

**Table 4:** Association between heavy alcohol consumption and sociodemographic—health determinants.

	*N* (%)	OR1	OR2
*Sex*			
Male	411 (16.95%)	1.0	1.0
Female	210 (8.51%)	**0.46 (0.38–0.54)**	**0.56 (0.46-0.69)**

*Age group*			
18–29	182 (14.85%)	1.0	1.0
30–39	143 (13.86%)	0.93 (0.73–1.17)	0.99 (0.74–1.31)
40-49	128 (13.70%)	0.91 (0.72–1.17)	0.96 (0.70–1.33)
50–59	104 (12.97%)	0.86 (0.66–1.12)	0.88 (0.61–1.25)
60–70	64 (7.11%)	**0.44 (0.33–0.6)**	**0.44 (0.27–0.72)**

*Marital status*			
Married	334 (11.15%)	1.0	1.0
Never-married	233 (16.11%)	1.15 (0.90–1.47)	**1.52 (1.17**–**1.99)**
Divorced	44 (18.33%)	**1.84 (1.30–2.61)**	**1.51 (1.04–2.19)**
Widowed	10 (4.69%)	0.58 (0.30–1.11)	0.56 (0.28–1.11)

*Educational level*			
None or primary	117 (12.63%)	1.0	1.0
Lower secondary	121 (15.18%)	0.89 (0.67–1.19)	0.77 (0.56–1.04)
Upper secondary	297 (12.65%)	**0.59 (0.45–0.69)**	**0.59 (0.44–0.79)**
Technical tertiary	44 (10.02%)	**0.47 (0.31–0.69)**	**0.46 (0.30–0.70)**
University	42 (10.94%)	**0.56 (0.38–0.83)**	**0.64 (0.41–0.98)**

*Employment status*			
Full or part-time job	435 (14.91%)	1.0	1.0
Looks after home	44 (6.37%)	**0.67 (0.47–0.96)**	0.72 (0.50–1.05)
Unemployed	36 (19.57%)	**1.51 (1.02–2.22)**	1.17 (0.78–1.76)
Retired	48 (8.32%)	**0.67 (0.46–0.96)**	1.04 (0.67–1.60)
Other/inactivity status	58 (11.09%)	**0.69 (0.51–0.94)**	0.79 (0.57–1.09)

*Residency*			
Urban	270 (10.07%)	1.0	1.0
Semi-urban	92 (15.16%)	**1.60 (1.24–2.07)**	**1.59 (1.22–2.07)**
Rural	259 (16.14%)	**1.74 (1.45–2.09)**	**1.72 (1.41–2.09)**

*Presence of financial difficulties*			
No	372 (11.33%)	1.0	1.0
Yes	249 (15.46%)	**1.55 (1.30–1.84)**	**1.28 (1.06–1.55)**

*Physical disease*			
No	78 (11.84%)	1.0	1.0
Yes	543 (12.82%)	1.15 (0.87–1.50)	1.15 (0.86–1.55)

*Smoking*			
No	234 (7.92%)	1.0	1.0
Yes	387 (19.98%)	**2.51 (2.10–3.00)**	**2.33 (1.94–2.81)**

*CIS-R score (categorical)*			
1–5	396 (11.4%)	**1.0**	**1.0**
6–11	126 (17.5%)	**1.99 (1.59–2.49)**	**1.79 (1.42–2.27)**
12–17	49 (14.8%)	**1.64 (1.18–2.27)**	1.34 (0.95–1.89)
≥18	50 (14%)	**1.77 (1.28–2.46)**	1.32 (0.93–1.88)
CIS-R score (continuous)		**1.03 (1.02–1.05)**	**1.02 (1.01–1.03)**

OR: Odds ratios from logistic regression analysis/CI: 95% confidence interval. OR1 = OR adjusted for sex and age. OR2 = fully adjusted OR (all presented variables are included concurrently). Bold values indicate statistically significant difference from reference category (*p* < 0.05).

**Table 5:** Association between alcohol use disorder and sociodemographic—health determinants.

	*N* (%)	OR1	OR2
*Sex*			
Male	124 (5.11%)	1.0	1.0
Female	30 (1.22%)	**0.23 (0.15–0.34)**	**0.23 (0.14-0.38)**

*Age group*			
18–29	51 (4.16%)	1.0	1.0
30–39	24 (2.33%)	**0.54 (0.33–0.90)**	**0.54 (0.31–0.97)**
40–49	35 (3.75%)	0.90 (0.58–1.41)	0.79 (0.43–1.44)
50–59	32 (3.99%)	0.98 (0.62–1.54)	0.75 (0.39–1.46)
60–70	12 (1.33%)	**0.32 (0.17–0.61)**	**0.19 (0.07–0.51)**

*Marital status*			
Married	75 (2.50%)	1.0	1.0
Never-married	58 (4.01%)	1.00 (0.63–1.62)	1.50 (0.87–2.56)
Divorced	17 (7.08%)	**3.15 (1.81–5.48)**	**2.44 (1.34–4.46)**
Widowed	4 (1.88%)	1.56 (0.54–4.48)	1.55 (0.51–4.75)

*Educational level*			
None or primary	34 (3.67%)	1.0	1.0
Lower secondary	35 (4.39%)	0.78 (0.47–1.31)	0.72 (0.42–1.23)
Upper secondary	74 (3.15%)	**0.44 (0.27–0.72)**	**0.50 (0.29–0.84)**
Technical tertiary	7 (1.59%)	**0.23 (0.10–0.54)**	**0.29 (0.12–0.73)**
University	4 (1.04%)	**0.17 (0.06–0.48)**	**0.26 (0.08–0.78)**

*Employment status*			
Full or part-time job	107 (3.67%)	1.0	1.0
Looks after home	7 (1.01%)	0.82 (0.35–1.96)	1.07 (0.43–2.65)
Unemployed	10 (5.43%)	1.92 (0.97–3.79)	1.17 (0.56–2.44)
Retired	10 (1.73%)	0.53 (0.25–1.10)	0.84 (0.35–1.97)
Other/inactivity status	20 (3.82%)	1.17 (0.70–1.97)	1.25 (0.73–2.16)

*Residency*			
Urban	66 (2.46%)	1.0	1.0
Semi- urban	20 (3.29%)	1.34 (0.80–2.24)	1.25 (0.73–2.14)
Rural	68 (4.24%)	**1.76 (1.25–2.49)**	**1.58 (1.08–2.30)**

*Presence of financial difficulties*			
No	90 (2.74%)	1.0	1.0
Yes	64 (3.97%)	**1.61 (1.16–2.25)**	1.01 (0.71–1.46)

*Physical disease*			
No	31 (4.40%)	1.0	1.0
Yes	123 (2.91%)	**2.31 (1.48–3.61)**	**2.12 (1.31–3.43)**

*Smoking*			
No	31 (1.05%)	1.0	1.0
Yes	123 (6.35%)	**5.07 (3.39–7.60)**	**4.59 (3.02–6.99)**

*CIS-R score (categorical)*			
0–5	73 (2.10%)	1.0	1.0
6–11	39 (5.47%)	**3.59 (2.39–5.41)**	**3.22 (2.1–4.95)**
12–17	22 (6.63%)	**4.53 (2.73–7.53)**	**3.28 (1.92–5.60)**
≥18	20 (5.62%)	**4.84 (2.84–8.26)**	**3.11 (1.74–5.53)**
CIS-R score (continuous)		**1.08 (1.06–1.10)**	**1.06 (1.04–1.08)**

OR: Odds ratios from logistic regression analysis/CI: 95% confidence interval. OR1 = OR adjusted for sex and age. OR2 = Fully adjusted OR (all presented variables are included concurrently). Bold values indicate statistically significant difference from reference category (*p* < 0.05).

**Table 6:** Association between heavy alcohol consumption (HAC) and mental health disorders.

OR (95% CI)	% with comorbid disorder	OR1	OR2
*Depression*			
No HAC	2.9%	1.0	1.0
HAC	2.9%	1.24 (0.75–2.07)	1.05 (0.62–1.79)

*Generalized anxiety disorder*			
No HAC	4.0%	1.0	1.0
HAC	4.7%	1.46 (0.97–2.21)	1.30 (0.86–1.98)

*Panic disorder*			
No HAC	1.8%	1.0	1.0
HAC	2.3%	1.52 (0.85–2.73)	1.37 (0.75–2.48)

*Obsessive-compulsive disorder*			
No HAC	1.7%	1.0	1.0
HAC	1.8%	1.24 (0.65–2.37)	1.10 (0.57–2.13)

*Phobic disorder*			
No HAC	2.6%	1.0	1.0
HAC	3.2%	1.55 (0.94–2.53)	1.37 (0.83–2.28)

*Mixed anxiety depressive disorder*			
No HAC	2.5%	1.0	1.0
HAC	3.7%	**1.77 (1.11–2.82)**	**1.72 (1.07–2.78)**

OR: Odds ratios from logistic regression analysis/CI: 95% confidence interval. OR1 = OR adjusted for sex and age. OR2 = OR adjusted for sex and age, marital status, educational level, employment status, financial difficulties, urbanicity/rurality of residency, smoking, and presence of physical disease. Bold values indicate statistically significant difference from reference category (*p* < 0.05).

**Table 7:** Association between alcohol use disorder (AUD) and mental health disorders.

	% with comorbid disorder	OR1	OR2
*Depression*			
No AUD	2.9%	1.0	1.0
AUD	4.6%	**2.24 (1.01–4.95)**	1.54 (0.67–3.54)

*Generalized anxiety disorder*			
No AUD	4%	1.0	1.0
AUD	7.8%	**2.96 (1.59–5.54)**	**2.42 (1.27–4.59)**

*Panic disorder*			
No AUD	1.8%	1.0	1.0
AUD	5.2%	**4.35 (2.02–9.38)**	**3.76 (1.71–8.28)**

*Obsessive-compulsive disorder*			
No AUD	1.7%	1.0	1.0
AUD	2.6%	2.01 (0.72–5.66)	1.60 (0.56–1.45)

*Phobic disorder*			
No AUD	2.5%	1.0	1.0
AUD	5.8%	**3.34 (1.63–6.84)**	**2.73 (1.31–5.73)**

*Mixed anxiety depressive disorder*			
No AUD	2.6%	1.0	1.0
AUD	3.9%	1.87 (0.80–4.38)	1.65 (0.70–3.92)

OR: Odds ratios from logistic regression analysis/CI: 95% confidence interval. OR1 = OR adjusted for sex and age. OR2 = OR adjusted for sex and age, marital status, educational level, employment status, financial difficulties, urbanicity/rurality of residency, smoking, and presence of physical disease. Bold values indicate statistically significant difference from reference category (*p* < 0.05).

## Data Availability

The data used to support the findings of this study are available from the corresponding author upon request.
